# Risk of fracture following gastric surgery for benign and malignant conditions: A study level pooled analysis of population-based cohort studies

**DOI:** 10.3389/fonc.2022.1001662

**Published:** 2022-11-21

**Authors:** Qiuping Zou, Chao Wei, Zhuo Shao, Hao Wang, Zhihong Xiao, Lixing Cao, Zubing Mei, Wei Zhao, Zhi Jiang, Zhiqiang Chen

**Affiliations:** ^1^ Department of Perioperative Research Center of Chinese Medicine, The Second Affiliated Hospital of Guangzhou University of Chinese Medicine, Guangzhou, China; ^2^ Emergency Department, Dongguan People’s Hospital, Dongguan, China; ^3^ Department of Spine Surgery, The First Affiliated Hospital of Fujian Medical University, Fuzhou, Fujian, China; ^4^ Department of Spine Surgery, National Regional Medical Center, Binhai Campus of The Affiliated Hospital, Fujian Medical University, Fuzhou, China; ^5^ Department of General Surgery, Changhai Hospital, Naval Medical University, Shanghai, China; ^6^ Department of Colorectal Surgery, Changhai Hospital, Naval Medical University, Shanghai, China; ^7^ Department of Spine Surgery, The Second Affiliated Hospital, University of South China, Hengyang, Hunan, China; ^8^ Department of Anorectal Surgery, Shuguang Hospital Affiliated to Shanghai University of Traditional Chinese Medicine, Shanghai, China; ^9^ Anorectal Disease Institute of Shuguang Hospital, Shanghai, China; ^10^ Department of General Surgery, Putuo District Central Hospital, Shanghai University of Traditional Chinese Medicine, Shanghai, China

**Keywords:** bone fracture, gastric cancer, surgery, cohort study, pooled analysis

## Abstract

**Background:**

Metabolic changes may occur following gastric surgery, which has been reported to contribute to bone loss, osteoporosis and even bone fracture. However, the evidence regarding the relationship between gastric surgery for benign and malignant conditions and risk of fracture is controversial. This study was conducted with the aim to evaluate whether gastric surgery is associated with a high risk of fracture.

**Methods:**

Major electronic databases were searched from inception through October 2021 for population-based cohort studies investigating the associations between gastric surgery (including bariatric gastric surgeries and surgeries for gastric benign and malignant gastric tumors) and risk of fracture compared with controls. Pooled relative risks (RRs) with 95% confidence intervals (CIs) were derived using the random-effects Mantel–Haenszel model. Multiple subgroup analyses and sensitivity analyses were carried out to test sources of heterogeneity stratified by various study characteristics and the robustness of the results.

**Results:**

A total of 14 studies comprising 693134 individuals were identified for analysis. The RR for the risk of fracture in people undergoing gastric surgery was 1.45 [95% confidence interval (CI) 1.23 - 1.72; I^2 =^ 95.8%; P < 0.001] compared with that in control populations, among which the fracture sites of upper limb, spine, lower limb, pelvis and hip showed consistent significant results (all P < 0.05), whereas nonsignificant associations was noted for other fracture sites. Significant associations were also observed for patients having total or subtotal gastrectomy (RR 2.22, 95% CI 1.66 to 3.00), gastric bypass (RR 1.48, 95% CI 1.26 to 1.74), and a similar trend was observed for preserved passage procedures (including sleeve gastrectomy, gastric banding, vertical banded gastroplasty and other procedures that preserved the passage through the duodenum and proximal small bowel, in contrast to gastric bypass), though the difference did not reach statistically significant (RR 1.10, 95% CI 0.95 to 1.26). An evident increased risk in the age range from 40-59 years was observed (40-49 years: RR 1.36, 95% CI 1.19-1.55; 50-59 years: RR 2.48, 95% CI 1.58-3.90).

**Conclusion:**

From this large pooled analysis of population-based cohort studies, evidence supports that fracture risk is increased in gastric surgery survivors compared with the control population. Early prevention and effective intervention strategies of bone fracture should be taken from clinicians and health policy makers.

**Clinical Trial Registration:**

PROSPERO (https://www.crd.york.ac.uk/prospero/display_record.php?RecordID=291394), identifier CRD42021291394

## Highlights

- People previously undergoing gastric surgery are subsequently at higher risk of fracture than control individuals.
- The highest fracture risk was seen after total or subtotal gastrectomy and gastric bypass.
- Increased risk for fractures was seen in the upper limb, spine, lower limb, pelvis and hip.
- Early prevention and effective intervention of bone fracture should be taken from clinicians in gastric surgery survivors.

## Introduction

Bone fracture is a major public health problem worldwide, which causes a heavy economic burden and seriously affects the quality of life of the middle-aged and elderly adults. With the high rate of disability, fracture is also a major cause of premature death (1, 2). Although the global age-standardized incidence rate for fracture and low bone mineral density (BMD) decreased slightly from 1990 to 2019, the absolute burden still increased significantly (3, 4). Older age and women gender seem to be two risk factors for fracture and low BMD. Studies have found that the global incidence of fractures in women is higher than that in men over the age of 64 (3). In Western countries, 1 in 3 women and 1 in 5 men may have osteoporotic fractures after the age of 50 (5). In China, women are reported to have a higher risk of developing low BMD than men (4).

Gastric cancer, as one of the top burdensome cancers globally, represents the second commonest cause of cancer death globally. Surgical treatment remains the cornerstone of cancer cure and palliation (6). In addition, with the increase in overweight and obese people, bariatric surgery has also become one of the most commonly performed gastrointestinal surgeries globally (7). For these benign and malignant conditions, the two most common types of gastric surgery are gastrectomy for benign and malignant gastric lesions, and various weight loss operations. The reported incidence of bone fracture in these patients following gastric surgery ranged from 20-40 per 1000 person-years (8, 9). The possible mechanism of fracture following this kind of surgery is that these operations can lead to endocrine changes and weight loss, which contributes to bone loss (10, 11). Weight loss can also cause the decrease of bone mineral density (BMD), and consequently, the risk of bone fracture increases (12). A number of studies have reported that upper gastrointestinal surgery, such as gastrectomy for gastric tumors and bariatric surgery, is significantly associated with osteoporotic fractures (13–17). However, most of these studies were hospital-based cohorts, case-control and uncontrolled cross-sectional studies (18–20), lack of population-based longitudinal cohort studies and large sample prospective studies. Therefore, the evidence of whether gastric surgery leads to an increased risk of fracture is still insufficient.

The inconsistent results of these studies prompted us to comprehensively assess the associations between gastric surgery and subsequent fracture risk through a systematic review. Moreover, we tried to explore the moderators, including study design, sample size, geographical regions, patient age, control population, fracture site, risk of bias, measurement of association, adjusted variables, and surgery type.

## Methods

This study is reported in accordance with the Preferred Reporting Items for Systematic Reviews and Meta-Analyses (PRISMA) (21) and Meta-analysis of Observational Studies in Epidemiology (MOOSE) guidelines (22), the protocol of which has been prospectively registered at PROSPERO (CRD42021291394) (https://www.crd.york.ac.uk/prospero/display_record.php?RecordID=291394).

### Search strategy and selection criteria

We developed the search strategies for PubMed, EMBASE, and Cochrane Central Register of Controlled Trials without language restriction for original peer-reviewed articles published before October 31, 2021 investigating the associations between gastric surgery (including bariatric gastric surgeries and surgeries for gastric benign and malignant gastric tumors) and risk of fracture compared with controls. Terms related to the three primary concepts (gastric surgery, fracture, and cohort study) were searched both as MeSH (Pubmed/Cochrane) terms or Emtree (Embase) terms and as text words. Full details for the complete search strategies and the search terms are provided in **Supplementary Methods**. Cross-referencing the bibliographies of the selected references was also conducted to identify additional relevant publications. When multiple publications from the same cohort were identified, we included data from the most recent publication or summarized a set of most comprehensive and updated data from all relevant publications. After screening all titles and abstracts for the remaining citations, we obtained full-text citations to determine eligibility. The whole literature screening process was performed by two reviewers independently. Conflicts were resolved through group discussion until consensus was reached. If necessary, disagreements were resolved with consultation of a third reviewer.

### Eligibility criteria

Studies were deemed appropriate for entry into the meta-analysis if they met the following inclusion or exclusion criteria: (1) study design: prospective/retrospective population-based cohort study; (2) participant: individuals previously underwent gastric surgery including bariatric surgery, total or subtotal gastrectomy for gastric lesions; (3) control: general populations having no history of gastric surgery matched or unmatched by demographic characteristics (4) the measure of association: studies reporting estimates including relative risk (RR), hazard ratio (HR), standardized incidence ratio (SIR) or incidence rate ratio (IRR) with corresponding 95% CIs that could be converted to the risk ratios. Cross-sectional studies, hospital-based or community-based observational studies and those providing inadequate data to generate precise estimates of the association between gastric cancer surgery and risk of fracture were all excluded. In addition, studies reported outcome of fracture resulting from metastatic cancer with bone localization, malnutrition/cachexia and bone loss due to the primary cancer were also excluded.

### Study selection, data collection, and data extraction

Three authors compiled a piloted data extraction template and independently extracted data from each included study. In case of any discrepancies, discussion was initiated or the opinion of a senior author was requested. Several fields of general data were then extracted from each paper and entered into the data extraction template: first author of the study, publication year, study design, geographical region, study period, observation period, population sample size, participants’ mean or median age, control population, method of diagnosis of the cohort, outcome ascertainment, the main result of the study and measure of associations.

### Quality assessment

For observational cohort studies, we used items from the Newcastle-Ottawa Scale (NOS) to evaluate methodological quality (23), with the primary aim to evaluate the representativeness of the population, selection of the cohorts and controls, ascertainment of exposure and outcomes and adequacy of follow-up. As was previously reported, an NOS score of 8-9 represented low risk of bias, and a score of 6-7 or less high risk of bias.

### Statistical analysis

Statistical analyses were undertaken using STATA Statistical Software (version 14.0; Stata Corporation, College Station, TX, USA). The pooled RR of fracture for people in the gastric surgery survivors compared with those in the general population or nonsurgery controls was the primary outcome measure. To account for the anticipated heterogeneity across studies, we employed the DerSimonian and Laird random effects meta-analysis to synthesize results (RRs with their corresponding 95% CIs) (24). Because the absolute risk of fracture was relatively low, the RR in cohort studies mathematically approximated the OR and other risk esimates; therefore, we reported all results as RRs in our analysis (25). Generally, we selected the maximally adjusted RRs to pool the results when various risk estimates with several adjustments were reported in a study. If the included studies did not provide RR for the association between gastric surgery and risk of fracture, we would try to calculate indirectly based on the given information (data or curves) in the original study using the method as previously reported by Parmar et al. (26). We used Cochran’s Q-statistic to test for between-study heterogeneity, and the I^2^ statistic was used to quantify the amount of between-study heterogeneity (27). To further explore the sources of heterogeneity, we carried out multiple subgroup analysis when two or more datasets per subgroup were available for the given analysis in term of study design, study populations, comparisons exposures, outcome measurements and risk of bias in all included studies. Besides, publication bias was tested by funnel plot symmetry combined with Egger’s test to explore small study effects (28). If publication bias was found existence, we would apply a Duvall and Tweedle trim-and-fill method to adjust for risk estimates (29). Sensitivity analysis was performed to test the relative influence of individual cohort on the combined results. All statistical tests were 2-sided and P values of <0.05 were considered statistically significant.

## Results

### Literature search and study characteristics

**Figure 1:**
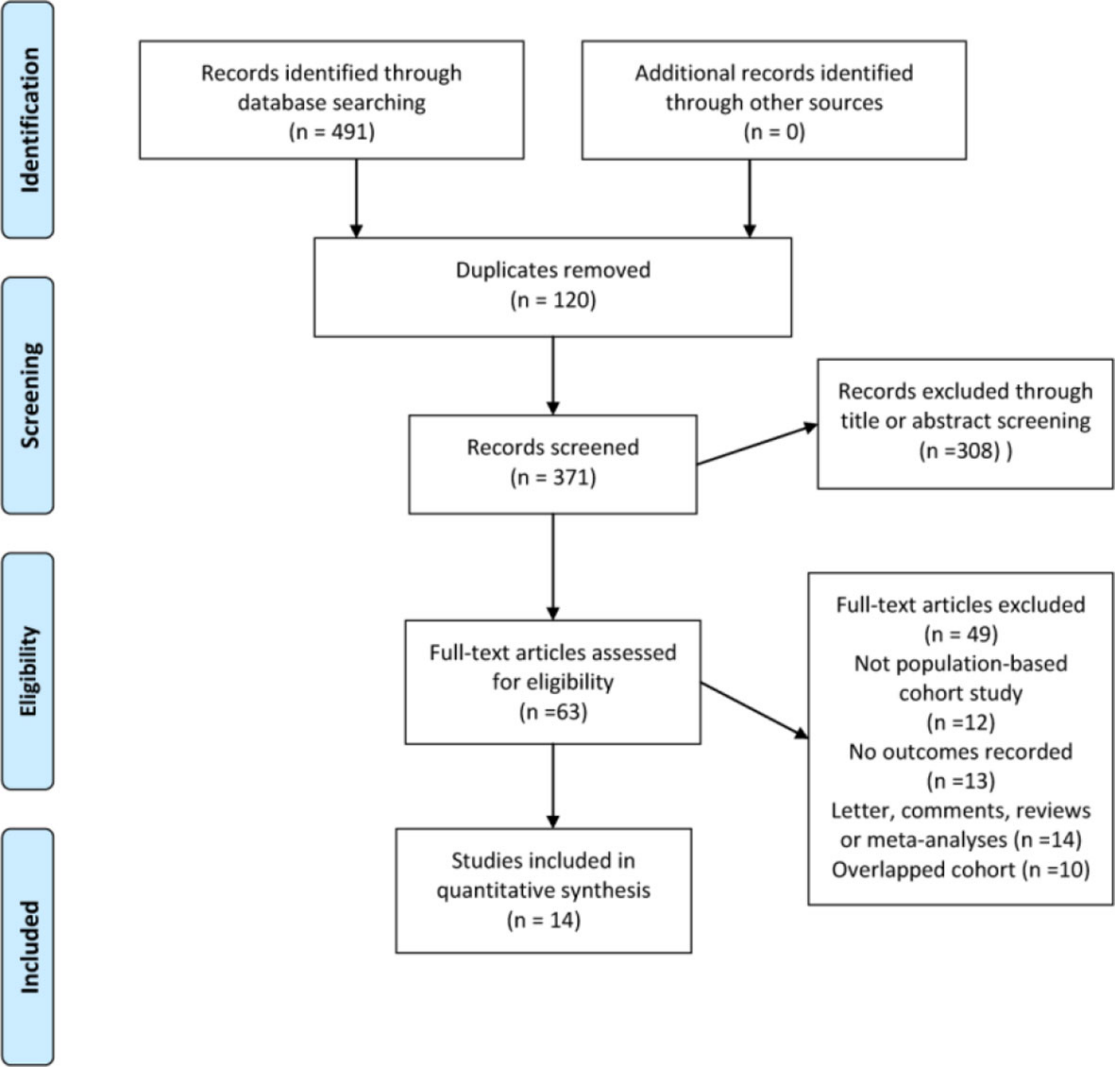
Flow diagram of literature screening according to PRISMA and MOOSE guidelines.

Our literature search identified 491 eligible citations, 120 of which were excluded due to duplication. A further 308 were subsequently excluded based on title and abstract review, yielding 63 citations for full-text review. Because of a lack of outcome data, non-population-based cohort study design, reviews and meta-analyses without original data, a total of 14 studies comprising 693134 individuals satisfied the inclusion criteria and were eligible to be included in the final meta-analysis and quality assessment (**Figure 1**). **Table 1** presents the sociodemographic and clinical characteristics of the 14 included studies (13, 15–17, 30–39).

**Table 1:** Baseline characteristics of studies included in the analysis of associations of gastric surgery with subsequent fracture risk.

Study	Year	Study design		Geographic region	Observation period	Population size, n	Participants’ mean age, years	Control population
Robinson et al.	2021	Retrospective popultion-based cohort study and popultion-based nested case-control study		UK	1997-2018	5487	40.7	Non-surgical self-controls
Chin et al.	2020	Retrospective popultion-based cohort study		China, Taiwan	2003-2008	5681	Bariatric surgery group 31.04; General population 32.17	Non-surgical patients;General population
Paccou et al.	2020	Retrospective popultion-based cohort study		France	2010-2014	81, 984	49.1	Obese population matched by age, sex, Charlson comorbidity index, year of inclusion, and class of obesity
Khalid et al.	2020	Retrospective multicenter popultion-based cohort study		USA	2004-2014	49113	NR	Matched bariatric surgery-eligible individuals who did not undergo bariatric surgery
Axelsson et al.	2018	Nationalwide retrospective cohort study		Sweden	1987-2014	77942	Diabetes: 47.3; Non-diabetes: 39.4	Propensity score matching generated well-balanced control groups for the obese patients both with and without diabetes
Rousseau et al.	2016	Population-based retrospective, nested case-control study		Canada	2001-2014	139436	42.6	Non-obese people of the same age ( ± 3 years) and sex
Lu et al.	2015	Nationalwide retrospective cohort study		China, Taiwan	2001-2009	7091	31.9	5027 non-surgery obese patients, using propensity score matching accounting for age, sex, Charlson Comorbidity Index, diabetes, hypertension, hyperlipidemia and the year morbid obesity was diagnosed
Douglas et al.	2015	Population-based observational retrospective cohort study		UK	From initial to 2014	7764	45	Non-surgery individuals from the CPRD matched with up to five of these individuals, matching on age, sex, general practice, and presence in the CPRD on the date bariatric surgery was recorded
Nakamura et al.	2014	Population-based retrospective cohort study		USA	1985-2004	258	44	Age and sex matched non surgery population
Lalmohamed et al.	2012	Population based retrospective cohort study.		UK	1987-2010	12521	Bariatric surgery 44.6; Matched controls 44.9	Matched by age, sex, practice, year, and body mass index
Melton III et al.	1999	Population based retrospective cohort study.		USA	1956-1985	438	56.6	NR
Shin et al.	2019	Nationalwide retrospective cohort study		Korea	2004-2012	266, 358	58.4	Noncancer control population matched for age, sex, residence, income, and disability
Iki et al.	2019	Population-based prospective cohort study		Japan	NR	1985	≥65	Male population with no history of gastrectomy
Seo et al.	2019	Nationalwide retrospective cohort study		Korea	2008-2010	37, 076	63.4	NR

Study	Surgical procedures performed		Method of Diagnosis				Results	Measure of associations
			Cohort ascertainment		Outcome ascertainment					
Robinson et al.	Partition surgeries (42.4%); gastric bypass surgeries (35.0%)		READ codes or hospital records using HES OPCS-4 codes.		Primary care records (READ codes) or an updated, for completeness, version of a previously validated list of READ codes.		The risk of fractures was elevated following bariatric surgery.	IRR
Chin et al.	Bariatric surgery (detailed type of surgery not recorded)		ICD-9 codes		ICD-9-CM		Bariatric surgery may decrease the risk of non-traffic accident–related fractures	HR
Paccou et al.	Bariatric surgeries including gastric bypass, sleeve gastrectomy, gastric banding, vertical banded gastroplasty		CCAM codes		ICD-10 codes		The risk of major osteoporotic fracture was significantly higher in the surgical group than in the matched obese controls.	HR
Khalid et al.	Bariatric surgeries including Roux-en-Y gastric bypass (RYGB) or sleeve gastrectomy (SG) (1:1)		ICD-9 codes		ICD-9 codes		Bariatric surgery was associated with a reduced risk of fracture in bariatric surgery–eligible patients.	OR
Axelsson et al.	Gastric bypass surgery		ICD-10 codes		ICD-10 codes		Gastric bypass surgery is associated with an increased fracture risk.	HR
Rousseau et al.	Adjustable gastric banding (n=3887); sleeve gastrectomy (n=2554); Roux-en-Y gastric bypass (n=873); biliopancreatic diversion (n=1986)		ICD-9/10 codes		ICD-9/10 codes		Patients undergoing bariatric surgery were more likely to have fractures than were obese or non-obese controls	RR
Lu et al.	Malabsorptive procedures (mainly gastric bypass) (289); restrictive procedures (mainly sleeve gastrectomy) (1775).		ICD-9 codes		ICD-9 codes		Bariatric surgery was significantly associated with an increased risk of fractures.	HR
Douglas et al.	Gastric band (47.1%); gastric bypass (36.6%); sleeve gastrectomy (15.8%).		CPRD read code		CPRD read code		No association was detected between bariatric surgery and fractures.	HR
Nakamura et al.	Gastric bypass (mainly Roux-en-Y gastric bypass) (94%)		Local medical care record identified through a centralized index to the diagnoses and surgical procedures		The original clinical history and radiologist’s report		Bariatric surgery is associated with an increased risk of fracture.	SIR
Lalmohamed et al.	Gastric banding(60%); Roux-en-Y gastric bypass (29%)		CPRD read code		CPRD read code		Bariatric surgery does not have a significant effect on the risk of fracture.	RR
Melton III et al.	Total or subtotal gastrectomy		Medical record		Radiologist’s report		The risk of osteoporotic fractures was significantly increased among patients operated for peptic ulcers.	SIR
Shin et al.	Subtotal gastrectomy (76.4%); total gastrectomy (23.6%)		ICD-10 codes		ICD-10 codes		Gastric cancer survivors who underwent gastrectomy had an increased osteoporotic fracture risk than did matched controls.	HR
Iki et al.	Gastrectomy (detailed total or partial resection not known)		Follow-up surveys		Diagnosed by a medical doctor with radiographs.		History of gastrectomy was associated with increased fracture risk in community-dwelling elderly Japanese men.	HR
Seo et al.	Subtotal gastrectomy (76.7%); total gastrectomy (23.3%)		ICD-10 codes		ICD-10 codes		Osteoporotic fracture incidences is high in patients within a relatively short timeframe after gastrectomy for stomach cancer	HR

CCAM, classification commune des actes médicaux; CPRD, Clinical Practice Research Datalink; HES, hospital episode statistics; HR, hazard ratio;

ICD, International Classification of Diseases; IRR, incidence rate ratio; NR, not reported; OPCS, Operating Procedure Codes; OR, odd ratio; RR, relative risk; SIR, standardised incidence ratio.

Of the studies published between 1999 and 2021, 4 studies were conducted in the United States (32, 34, 37, 39), 5 in Europe (13, 15, 30, 36, 38) and 5 in Asia (16, 17, 31, 33, 35). Most reports were retrospective cohort studies using population-based or national wide databases as data sources. Studies ranged in size from 258 to 266358 participants with a median sample size of 10143 (interquartile range, 5536-70735). The median follow-up duration was 4.5 years (range, 2.2-14.8 years). Nine studies enrolled individuals undergoing gastric surgery and control populations matched for at least five variables. Most studies ascertained the diagnosis and fracture outcome through medical records according to the ICD-9/10 codes.

### Methodological quality (risk of bias)

The overall risk of bias was moderate to high for all studies based on the NOS tool. Bias was frequently seen in term of adequacy of follow-up followed by selection of control cohort and comparability of cohorts. We found that two studies (13, 35) was judged low risk of bias in all domains with an NOS score of 9. The detailed rationale for the risk of bias assessment is present in **Table 2**.

**Table 2:** Methodological quality score of the included studies based on the Newcastle–Ottawa scale (NOS) tool.

Study	Year	Study design	Selection				Comparability	Exposure/Outcome			Total Score	Risk of bias
			Representativeness of cohort *	Selection of control cohort *	Ascertainment of exposure *	Outcome not present at start *	Comparability of cohorts **	Assessment of outcome *	Length of follow-up *	Adequacy of follow-up *	Total score 9*	
Robinson et al.	2021	Retrospective popultion-based cohort study and popultion-based nested case-control study	*		*	*	**	*	*		7	Moderate
Chin et.al.	2020	Retrospective popultion-based cohort study	*	*	*	*	**	*	*		8	Low
Paccou et.al.	2020	Retrospective popultion-based cohort study	*	*	*	*	**	*	*		8	Low
Khalid et.al.	2020	Retrospective multicenter popultion-based cohort study	*	*	*	*	*	*	*		7	Moderate
Shin et.al.	2019	Nationalwide retrospective cohort study	*	*	*	*	**	*	*		8	Low
Iki et.al.	2019	Population-based prospective cohort study	*	*		*	**	*	*		7	Moderate
Seo et.al.	2019	Nationalwide retrospective cohort study	*		*	*	**	*		*	7	Moderate
Axelsson et.al.	2018	Nationalwide retrospective cohort study	*	*	*	*	**	*	*	*	9	Low
Rousseau et.al.	2016	Population-based retrospective, nested case-control study	*	*	*	*	**	*		*	8	Low
Lu et.al.	2015	Nationalwide retrospective cohort study	*	*	*	*	**	*	*	*	9	Low
Douglas et.al.	2015	Population-based observational retrospective cohort study	*	*	*	*	**	*	*		8	Low
Nakamura et.al.	2014	Population-based retrospective cohort study	*	*	*	*	*	*	*		7	Moderate
Lalmohamed et.al.	2012	Population based retrospective cohort study.	*	*	*	*	**	*	*		8	Low
Melton III et.al.	1999	Population based retrospective cohort study.	*		*	*	*	*	*		6	Moderate

* represents one score and ** represents two scores.

### Associations between gastric surgery and the risk of fracture

**Figure 2:**
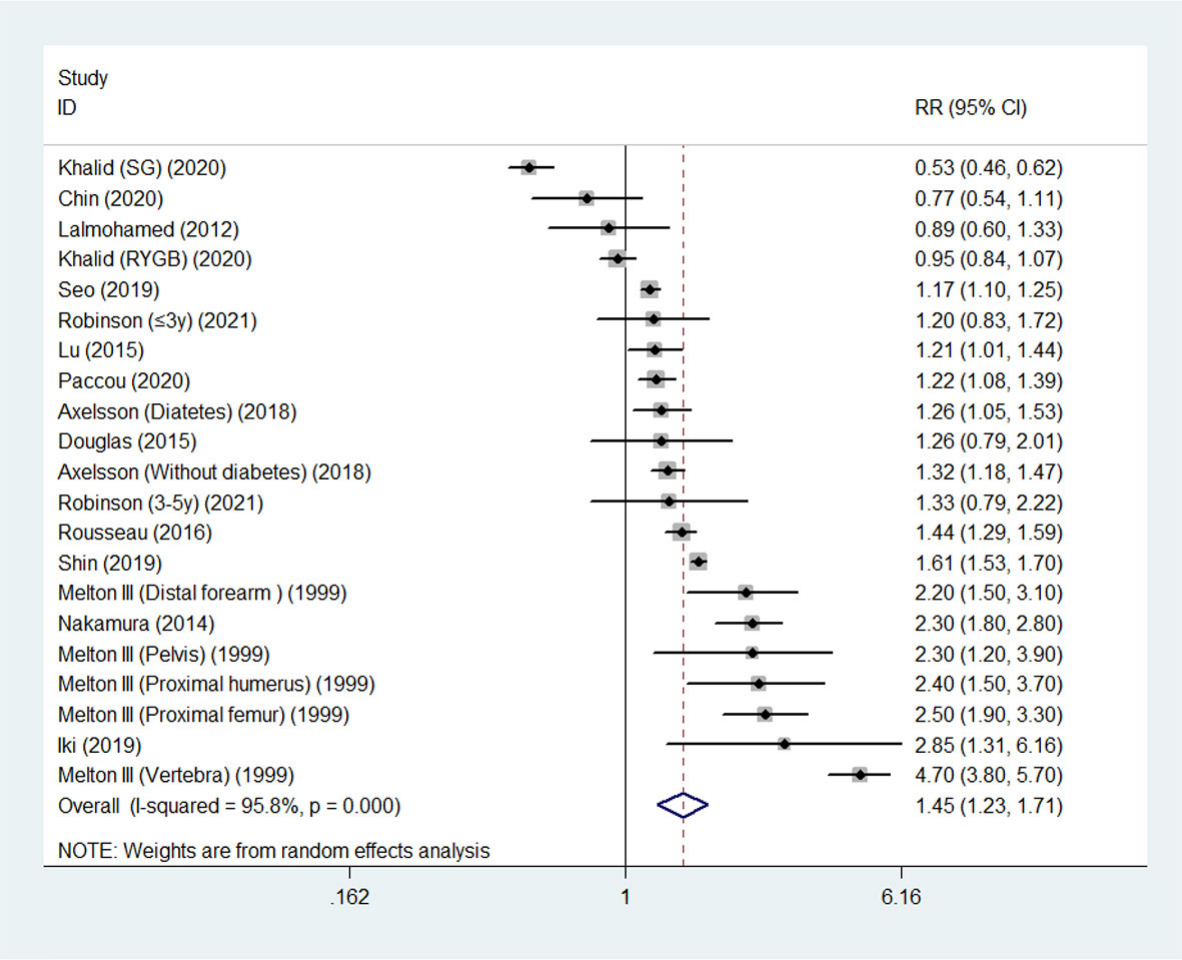
Relative risk (RR) for association between gastric surgery and fracture risk.

Overall, random-effects meta-analysis of the 14 studies showed that the summary RR of fracture reached 1.45 (95% CI, 1.23 - 1.72) in survivors following gastric surgery compared with control populations. We noted significant inter-study heterogeneity (I^2 =^ 95.8%; *P*< 0.001) (**Figure 2**).

### Subgroup analysis

Subgroup analyses indicated that gastric surgery was associated with an increased risk of fracture among survivors with an age range from 40 to 59 years (40-49 years, RR 1.36, 95% CI 1.19-1.55; 50-59 years, RR 2.48, 95% CI 1.58-3.90), while not among survivors ≥ 65 years (RR 1.67, 95% CI 0.71-3.94) or with an age range from 30 to 39 years (RR 1.00, 95% CI 0.65-1.54) (**Table 3**). For specific investigated fracture sites, it was found that significant associations were noted for risks of upper limb (shoulder, humerus, elbow, forearm, and wrist) fracture (RR 1.33, 95% CI 1.09 -1.63), spine fracture (RR 1.34, 95% CI 1.05-1.71), pelvis and hip fracture (RR 1.89, 95% CI 1.48-2.42) and lower limb fracture (RR 1.53, 95% CI 1.10-2.11) (**Table 4**). Significant associations were also observed for patients having total or subtotal gastrectomy (RR 2.22, 95% CI 1.66 to 3.00), gastric bypass (RR 1.48, 95% CI 1.26 to 1.74) (**Tables 3**, **4**), and a similar trend was observed for preserved passage procedures (including sleeve gastrectomy, gastric banding, vertical banded gastroplasty and other procedures that preserved the passage through the duodenum and proximal small bowel, in contrast to gastric bypass), though the difference did not reach statistically significant (RR 1.10, 95% CI 0.95 to 1.26). Furthermore, the increased fracture risk associated with gastric surgery was more evident among several other subgroups including in studies with different study design, sample size less than 10000, studies conducted in different geographical regions, with different measurement of associations, risk of bias and different degree of adjustment.

**Table 3:** Subgroup analyses for the effect of gastric surgery on risk of fracture.

Variables	RR	95% CI	I^2^ (%)	No. studies	*P* for interaction
Study design					0.060
Prospective cohort	2.85	1.31 to 6.18	–	1	
Retrospective cohort	1.43	1.21 to 1.69	95.9	13	
Sample size					<0.001
<10000	1.86	1.32 to 2.61	92.4	7	
≥10000	1.12	0.92 to 1.36	96.8	7	
Patient age					<0.001
30-39 years	1.00	0.65 to 1.54	78.9	2	
40-49 years	1.36	1.19 to 1.55	74.7	7	
50-59 years	2.48	1.58 to 3.90	95.5	2	
≥60 years	1.67	0.71 to 3.94	80.2	2
Geographical regions					0.034
USA	1.82	1.16 to 2.85	97.9	4	
Europe	1.27	1.01 to 1.61	0	5	
Asia	1.26	1.17 to 1.35	94.6	5	
Controls					0.128
Nonsurgery populations	1.42	0.40 to 5.07	88.8	2	
Matched populations	1.20	0.98 to 1.48	96.1	9	
Others	1.24	0.92 to 1.67	0	2	
Measurement					0.312
HR	1.27	1.11 to 1.46	89.9	8	
Others	1.62	1.11 to 2.36	97.2	6	
Risk of bias					<0.001
Low (≥8)	1.26	1.12 to 1.43	83.7	8	
Moderate (6-7)	1.72	1.22 to 2.44	97.2	6	0.047
Number of adjusted variables					<0.001
≥5 (Fully adjustment)	1.29	1.14 to 1.46	88.7	9	
<5 (Not fully adjustment)	1.64	1.02 to 2.62	97.4	5	
Total/subtotal gastrectomy					0.002
Yes	2.22	1.66 to 3.00	93.5	4	
No	1.14	0.94 to 1.39	96.7	10	
Surgery for benign or malignant conditions					
Benign condition	1.44	1.15 to 1.81	95.8	11	
Malignant condition	1.49	1.11 to 2.01	96.7	3	

CI, confidence interval; NA, not available; RR, relative risk.

**Table 4:** Subgroup analyses based on fracture site and surgery type.

Variable	RR	95% CI	I^2^ (%)	No. studies
**Fracture site**				
Skull/face	1.40	0.25 to 7.79	85	2
Upper limb (shoulder, humerus, elbow, forearm, and wrist)	1.33	1.09 to 1.63	81.6	6
Spine	1.34	1.05 to 1.71	26.6	5
Clavicle/scapula/sternum/rib	1.65	0.94 to 2.88	51.4	2
Pelvis and hip	1.89	1.48 to 2.42	29.4	6
Lower limb	1.53	1.10 to 2.11	86.2	4
**Surgery type**				
Preserved passage procedures (Such as sleeve gastrectomy, gastric banding and vertical banded gastroplasty)	1.10	0.95 to 1.26	55.6	4
Sleeve gastrectomy	1.08	0.92 to 1.28	38.1	3
Adjustable gastric banding	0.99	0.83 to 1.18	84.1	3
Others (Vertical banded gastroplasty or biliopancreatic diversion)	1.25	0.75 to 2.08	96	2
Gastric bypass	1.48	1.26 to 1.74	91.0	8
Total/subtotal gastrectomy	2.22	1.66 to 3.00	93.5	4

CI, confidence interval; RR, relative risk.

In addition, heterogeneity was high in the analysis of subgroups of studies conducted in the United States (I^2^ = 97.9%) and Asia (I^2^ = 94.6%), but was not detected in Europe (I^2^ = 0.0%). Similarly, heterogeneity was also significant in the subgroups of elder age (≥50 years) (I^2^ = 95.5% and 80.2%, respectively) and younger individuals (30-39 years) (I^2^ = 78.9%), fracture site of skull/face (I^2^ = 85.0%), upper limb (I^2^ = 88.8%) and lower limb(I^2 =^ 86.2%), and surgery type of gastric bypass (I^2^ = 91.0%) and adjustable gastric banding (I^2^ = 84.1%), but was slight or not detected in the subgroups of fracture site of spine (I^2^ = 26.6%), pelvis and hip (I^2^ = 29.4%), the subgroup with surgery type of sleeve gastrectomy (38.1%). These analyses indicated that geographic region, age, surgery type, and fracture site could be potential sources of heterogeneity. Moreover, the residual heterogeneity could originate from other variation in demographic variables among the individuals of each included study.

### Sensitivity analyses and publication bias

Using the leave-one-out sensitivity analyses, we further tested the stability of the result and indicated that no single study substantially altered the pooled risk estimates (lowest RR 1.35, 95% CI, 1.17-1.56; highest RR 1.53, 95% CI, 1.32-1.77) (**Supplementary Table S1** and **Supplementary Figure S1**). Visual inspection of the contour enhanced funnel plot indicated asymmetry, which implied evidence of publication bias (**Supplementary Figure S2**). Publication bias test found no missing studies in the funnel plot region, suggesting that publication bias was unlikely to be the main cause of plot asymmetry. Both Begg’s test and Egger’s test for small study effects were insignificant (*P*= 0.381 for Begg’s test and *P*= 0.764 for Egger’s test).The trim and fill method used to adjusted for publication bias did not lead to imputation of any hypothetical missing studies, and the risk estimate remained the same.

## Discussion

### Principal findings

In this pooled analysis of 14 population-based cohort studies, we found statistically significant increase in the risk of fracture for gastric surgery survivors compared to that for nonsurgery individuals. The results remained largely unchanged after adjustment for potential publication bias. Moreover, our results indicate that gastric surgery contributes to the future development of fracture especially for individuals with an age range from 40 to 59 years, fracture sites including upper limb, lower limb, pelvis and hip, and the results is constant across different geographical regions and other study features.

Based on the results of subgroup analyses, we found that the fracture risk was significantly increased for different types of gastric surgery including gastric bypass and total or subtotal gastrectomy. These findings appear reasonable, because these surgeries either divert ingested nutrients or reduce the volume of the stomach, which will have a significant impact on nutrient absorption in the stomach and duodenum, affecting bone metabolism and increasing the risk of fracture. Despite the fact that the fracture risk for preserved passage procedures was not statistically significant, we propose that larger prospective cohort studies be conducted to demonstrate the associations. We also found that the risk of fracture was higher in patients over the age of 40, which indicated that the effect of gastric surgery on gastric absorptive compensation was more obvious in older patients than in younger patients.

The results of our study are similar to and support previously findings from other published systematic reviews and meta-analyses, which also demonstrated the association between gastric surgery and subsequent risk of fracture (40–43). However, those four review articles only focused on obese patients undergoing specific bariatric surgery. Our study further extended the participants including all individuals receiving gastric surgery for gastric tumor or ulcer removal, and weight loss (bariatric surgery). Moreover, these four meta-analyses mostly used non-representative cohorts with relatively high risk of selection bias. To the best of our knowledge, this pooled analysis is the first and most comprehensive one involving high representative populations to meta-analyze the associations between previous gastric surgery and subsequent fracture risk from less biased population-based cohorts.

An evident increased risk in the age range from 40-59 years was observed, indicated by the summary RR through subgroup analyses stratified by patient age (**Table 3**). Though the hypothesis of this finding is not clear, the result should be further confirmed by large prospective cohort studies as there were few studies included for analyses with limited statistical power.

### Potential mechanisms

The potential mechanism underlying the gastric surgery-related increase in fracture risk is not so clearly demonstrated. Several possible theories have been proposed to explain this finding.

A significant metabolic change after gastric surgery is malabsorption of calcium and vitamin D (44). Due to low gastric acidity in the remnant stomach after gastrectomy or bariatric surgery, and there is no passage of nutrients through the duodenum in patients having had gastric bypass, calcium absorption will be reduced (45). Another possible cause of calcium deficiency is reduced food intake after gastric surgery (46). Other causes such as pancreatic exocrine dysfunction after gastric surgery or inactivation of lipase caused by bacterial overgrowth can also affect vitamin D absorption (17, 47). Secondly, inadequate dietary intake and changes in calcium and vitamin D metabolism can lead to secondary hyperparathyroidism (48, 49). Meanwhile, in order to maintain serum calcium levels, bone mass will decrease. Hyperparathyroidism can lead to adverse changes in the microstructure of cortical bone, which increases the risk of osteoporosis and fracture (50, 51).

Metabolism-related weight loss is the third potential cause of increased fracture risk in people after gastric surgery. Due to the changes in gastrointestinal anatomy after gastric surgery, gastrointestinal motility and function also change, resulting in irreversible functional changes such as dyspepsia and malabsorption (52, 53). Most of these patients will experience varying degrees of weight loss after operation (54). Weight loss can change the mechanical load bearing of human bones, which can increase the risk of fractures. In addition, bariatric surgery will lead to the reduction of a variety of hormones in the body, such as estrogen and insulin, thus affecting bone metabolism and aggravating bone loss (55, 56). It has been reported that weight loss after gastrectomy is the main factor aggravating bone loss (46, 57).

### Strengths and limitations

Compared to previous ones, this study has several important strengths. Firstly, the current study included the first and the largest sample to date using high representative population for pooled analysis, providing a comprehensive summary of the evidence on the association between of gastric surgery and subsequent fracture risk. Secondly, we comprehensively searched the relevant databases using sensitive search strategies, facilitating retrieval of as many relevant studies as possible globally. Thirdly, we only selected studies of representative national wide or population-based cohort and excluded studies of hospital/community-based cohort studies. The high-quality evidence makes the results more credible. Fourthly, we explored the sources of heterogeneity and impact of publication bias through the use of multiple approaches including subgroup analyses, sensitivity analyses, trim and fill analyses. Our findings confirmed that the main results were robust.

Nevertheless, several limitations are evident in our study. Firstly, a high degree of inter-study heterogeneity was found. Though multiple subgroup analyses were conducted, there was still considerable moderate to high between-study heterogeneity. Even so, the results of the subgroup analysis and sensitivity analysis are mostly consistent with the main result. Therefore, we believe that heterogeneity does not substantially affect the main findings to a great extent. However, one major concern was that we could not assess the effect of participants’ treatment with vitamin D and calcium supplements on the result of our findings due to the unavailability of such information from the majority of the included studies. Secondly, our findings are mainly based on a retrospective cohort study (19, 20), in which the design of the study may be subject to a variety of confounding factors and bias. However, the results of 9 from those 14 studies were obtained from fully matched covariates (≥5 adjusted variables) and compared with the non-surgery or general population (**Table 4**), indicating that this association was consistent among different clinical scenarios. Thirdly, we performed the pooled analysis based on the study level evidence. Therefore, we could not carried out more subgroup analyses (e.g. fracture time point following surgery) due to the lack of access to the individual patient data. Fourthly, nonsignificant risk estimates obtained from a few subgroup analyses may be due to the low statistical power caused by insufficient sample size. We advocate that high quality prospective cohort studies on this aspect be carried out in the future. Finally, we only included three major databases (PubMed, Embase and Cochrane Library) without involving the unpublished grey literature for analysis though they covered more than 90% of all citations.

### Implications

Despite all these limitations, the current study provides alarming clinical implication for risk of fracture in people undergoing gastric surgery, estimating a crude risk ratio for any fracture of 1.45 (95% CI, 1.23 - 1.72). Early prevention and timely interventions are of great clinical significance in the prevention and treatment of these high-risk individuals. Moreover, the increased risk of fracture should be also mentioned during the preoperative informed consent process. Additionally, there is need for better understanding of the pathophysiological mechanisms, basic research on hormonal and neuro-intestinal pathways responsible for decreased bone quality.

## Conclusions

In conclusion, this meta-analysis showed that individuals who underwent gastric surgery may have an increased risk of fracture. Based on the subgroup analysis results stratified by most baseline variables, it is found that the results are still consistent and biologically plausible. However, before we get a high level of evidence based on prospective large cohort studies to prove this relationship, we should still interpret the results very carefully.

## Data availability statement

The datasets presented in this study can be found in online repositories. The names of the repository/repositories and accession number(s) can be found in the article/**Supplementary Material**.

## Author contributions

Study concept and design: ZM, WZ, ZJ, ZC. Acquisition of data: QZ, CW, ZS, HW, ZM. Analysis and interpretation of data: CW, ZX, HW, ZM, WZ, ZJ, LC. Drafting of the manuscript: QZ, ZM. Critical revision of the manuscript for important intellectual content: All authors. Corresponding authors: ZM, WZ, ZJ, ZC.

## Funding

This study was supported by the Department of Science and Technology of Guangdong Province (No.2021A151511077) and the Traditional Chinese Medicine Bureau of Guangdong Province (No.20225012), and the Special Project for Clinical Research of Guangdong Provincial Hospital of Chinese Medicine (YN10101902) , the Guangdong Administration for Market Regulation (Guangdong Intellectual Property Administration) (No.〔2022〕158), the National Regional Traditional Chinese Medicine (Specialist) Clinic Construction ((2018)205), Scientific Research Project from the Education Department of Fujian Province(No.JAT190205) and Joint Funds for the Innovation of Science and Technology, Fujian Province (No.2020Y9112).

## Conflict of interest

The authors declare that the research was conducted in the absence of any commercial or financial relationships that could be construed as a potential conflict of interest.

## Publisher’s note

All claims expressed in this article are solely those of the authors and do not necessarily represent those of their affiliated organizations, or those of the publisher, the editors and the reviewers. Any product that may be evaluated in this article, or claim that may be made by its manufacturer, is not guaranteed or endorsed by the publisher.
