# Supplementary material for: Risk of fracture following gastric surgery for benign and malignant conditions: A study level pooled analysis of population-based cohort studies
Source: Front Oncol. 2022 Nov 21;12:1001662. doi: 10.3389/fonc.2022.1001662 (PMC9721391; doi:10.3389/fonc.2022.1001662)

Supplementary Data

Supplementary Methods

Text S1 Search strategy

Database: Pubmed from inception to Present> (Search date: October 31, 2021)

Search Strategy:

Fracture terms:

1 "Fractures, Bone"[Mesh]

2 fractur*[Title/Abstract]

3 or/1-2

Gastric Surgery terms:

4 "Gastrectomy"[Mesh]

5 "Bariatric Surgery"[Mesh]

6 (Gastrectomy or jejunoileal bypass OR vertical banded gastroplasty OR gastric bypass OR gastric bypass loop OR adjustable gastric band OR biliopancreatic diversion OR bariatric*)[Title/Abstract]

7 or/4-6

Study design terms:

8 "Retrospective Studies"[Mesh]

9 "Cohort Studies"[Mesh]

10 "Longitudinal Studies"[Mesh]

11 "Follow-Up Studies"[Mesh]

12 "Prospective Studies"[Mesh]

13 (cohort or longitudinal or followup or prospective*or retrospective* or database* or population* or follow up)[Title/Abstract]

14 "Registries"[Mesh]

15 (registry or registries) [Title/Abstract]

16 or/8-15

Final search results: Combining Fracture and Gastric Surgery and Study design:

19 3 and 7 and 16 (140)

Text S2 Search strategy

Database: Embase from inception to Present> (Search date: October 31, 2021)

Search Strategy:

Fracture terms:

1 'fracture'/exp

2 fractur*:ab,ti

3 or/1-2

Gastric Surgery terms:

4 'gastrectomy'/exp

5 'bariatric surgery'/exp

6 (Gastrectomy or jejunoileal bypass OR vertical banded gastroplasty OR gastric bypass OR gastric bypass loop OR adjustable gastric band OR biliopancreatic diversion OR bariatric*):ab,ti

7 or/4-6

Study design terms:

8 'retrospective study'/exp

9 'cohort analysis'/exp
10 'longitudinal study'/exp
11 'follow up'/exp
12 'prospective study'/exp
13 (cohort or longitudinal or followup or prospective*or retrospective* or database* or population* or follow up) :ab,ti
14 'register'/exp
15 (registry or registries) :ab,ti
16 or/8-15

Final search results: Combining Fracture and Gastric Surgery and Study design:

17 3 and 7 and 16 (350)

Text S3 Search strategy

Database: Cochrane Library from inception to Present> (Search date: October 31, 2021)

1 MeSH descriptor: [Fractures, Bone] explode all trees
2 (fractur*):ti,ab,kw (Word variations have been searched)
3 #1 or #2
4 MeSH descriptor: [Gastrectomy] explode all trees
5 MeSH descriptor: [Bariatric Surgery] explode all trees
6 (fractur*Gastrectomy or jejunoileal bypass OR vertical banded gastroplasty OR gastric bypass OR gastric bypass loop OR adjustable gastric band OR biliopancreatic diversion OR bariatric*):ti,ab,kw (Word variations have been searched)
7 #4 or #5 or #6
8 MeSH descriptor: [Retrospective Studies] explode all trees
9 MeSH descriptor: [Cohort Studies] explode all trees

10 MeSH descriptor: [Longitudinal Studies] explode all trees

11 MeSH descriptor: [Follow-Up Studies] explode all trees

12 MeSH descriptor: [Prospective Studies] explode all trees

13 (cohort or longitudinal or followup or prospective*or retrospective* or database* or population* or follow up):ti,ab,kw (Word variations have been searched)

14 MeSH descriptor: [Registries] explode all trees

15 (registry or registries):ti,ab,kw (Word variations have been searched)

16 #8 or #9 or #10 or #11 or #12 or #13 or #14 or #15

17 #3 and #7 and #15 (1)

Supplementary Table S1. Results of sensitivity analysis using the leave-one-out method.

Study ommited	RR	95% CI
Robinson (≤3y)	1.46	1.23-1.74
Robinson (3-5y)	1.46	1.23-1.73
Chin	1.49	1.26-1.77
Paccou	1.47	1.23-1.75
Khalid (SG)	1.53	1.32-1.77
Khalid (RYGB)	1.49	1.25-1.77
Shin	1.45	1.20-1.75
Iki	1.43	1.20-1.69
Seo	1.48	1.22-1.78
Axelsson (Diatetes)	1.46	1.23-1.74
Axelsson (Without diabetes)	1.46	1.22-1.75
Rousseau	1.45	1.22-1.74
Lu	1.47	1.23-1.75
Douglas	1.46	1.23-1.73
Nakamura	1.42	1.19-1.68
Lalmohamed	1.48	1.25-1.76
Melton III (Vertebra)	1.35	1.17-1.56
Melton III (Proximal humerus)	1.42	1.20-1.68
Melton III (Distal forearm)	1.42	1.20-1.69
Melton III (Pelvis)	1.43	1.21-1.69
Melton III (Proximal femur)	1.41	1.19-1.67

Supplementary Figure S1. Sensitivity analysis for association between gastric surgery and risk of fracture using the leave-one-out method.

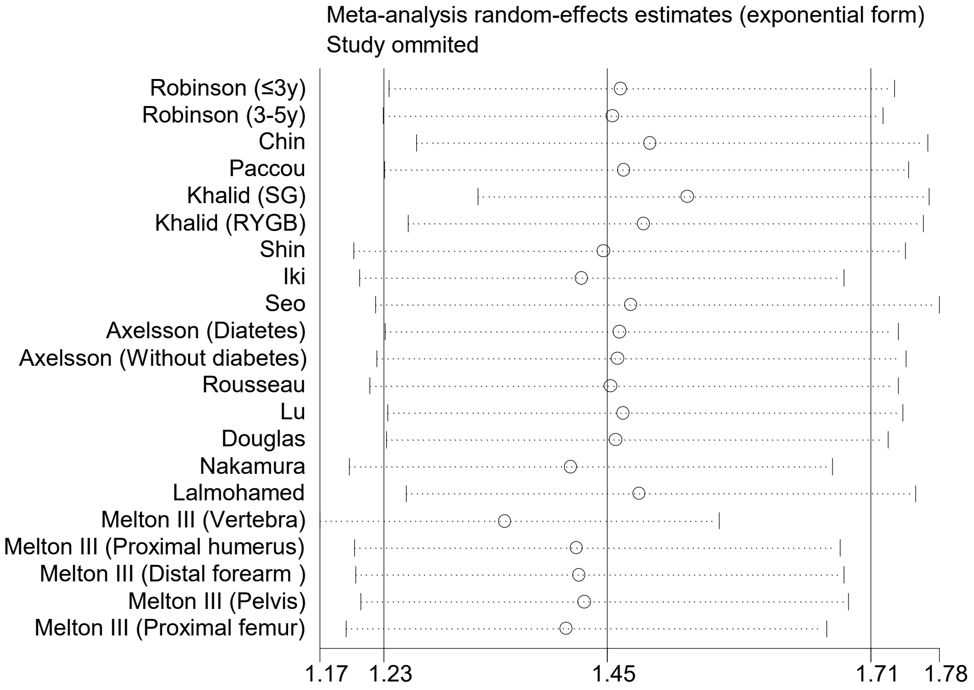

Supplementary Figure S2. Contour-enhanced funnel plot for meta-analysis of the association between gastric surgery and risk of fracture.

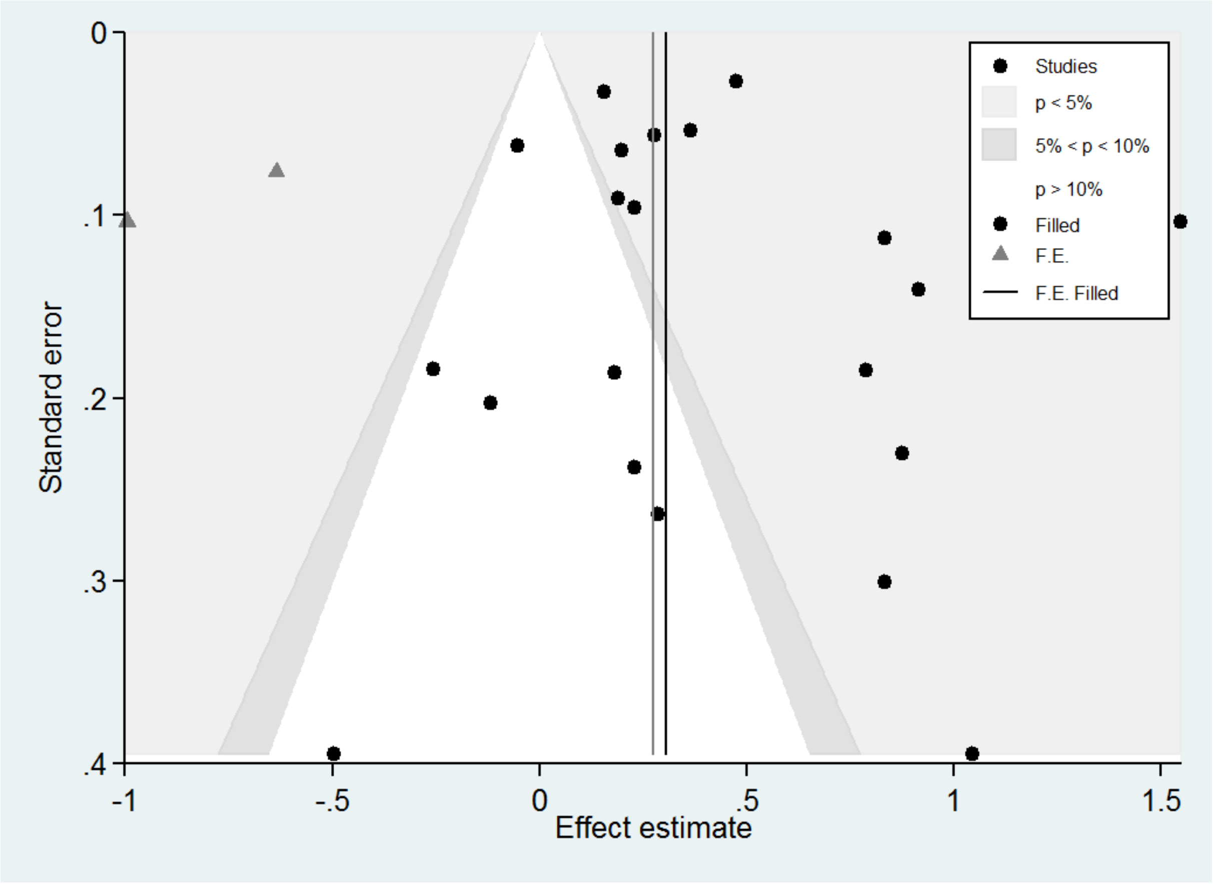

Supplement: Supplementary file 1 [file DataSheet_1.pdf]
